# Current and Emerging Treatments for Isolated Aortic Stenosis and Concomitant Mitral Stenosis: A Comprehensive Narrative Review

**DOI:** 10.3390/jcm15103674

**Published:** 2026-05-10

**Authors:** Kevin Martini, Salvatore Poddi, Alessio Rungatscher

**Affiliations:** Division of Cardiac Surgery, University of Verona Medical Center, 37126 Verona, Italy

**Keywords:** aortic stenosis, mitral stenosis, cardiac surgery, aortic valve replacement, multivalvular disease, transcatheter aortic valve replacement

## Abstract

Aortic stenosis (AS) and mitral stenosis (MS) are progressive valvular heart diseases associated with substantial morbidity and mortality once symptoms develop. Over the past decade, the management of isolated AS has undergone profound evolution, driven by refinements in surgical aortic valve replacement, the adoption of minimally invasive techniques, and the rapid expansion of transcatheter aortic valve replacement across all surgical risk categories. In contrast, patients with concomitant AS and MS represent a complex and understudied population, frequently excluded from randomized trials and only marginally addressed in contemporary clinical practice guidelines. The management requires individualized guideline-directed decision-making led by a multidisciplinary Heart Team. The paucity of high-quality data in combined AS–MS underscores the need for dedicated prospective studies and international registries. The aim of this narrative review is to describe current strategies to treat AS both when isolated and concomitant with MS. We also discuss the need for updated, specific guidelines.

## 1. Introduction

Aortic stenosis (AS) is the most prevalent valvular heart disease in developed countries and represents a growing public health challenge in aging populations. Degenerative calcific AS predominates in contemporary practice, with prevalence increasing sharply with age and exceeding 4–5% among individuals older than 65 years. Once symptoms develop (exertional dyspnea, angina, syncope), untreated severe AS is associated with a dramatic reduction in survival, underscoring the importance of timely intervention [1,2,3].

For decades, surgical aortic valve replacement (SAVR) has represented the cornerstone of treatment for severe symptomatic AS. SAVR provides immediate relief of left ventricular outflow tract obstruction (LVOTO), predictable hemodynamic improvement, and excellent long-term durability [4,5,6]. From a cardiac surgery perspective, an additional advantage of SAVR lies in the ability to address concomitant cardiac pathology—including coronary artery disease, ascending aortic disease, and additional valvular lesions—during a single operative session [1,2]. These attributes have historically established surgery as the reference standard against which emerging therapies are compared [4,5,7,8].

The introduction of transcatheter aortic valve replacement (TAVR) has fundamentally transformed the therapeutic landscape of AS. Initially developed for patients at prohibitive surgical risk, TAVR has demonstrated favorable outcomes in randomized trials and large registries, leading to its progressive adoption in intermediate- and low-risk populations [8,9,10]. Contemporary international guidelines now emphasize individualized decision-making based on patient age, life expectancy, anatomical suitability, and procedural risk, rather than surgical risk scores alone [1,2]. While TAVR offers clear advantages in selected patients (particularly reduced invasiveness and faster recovery), it also introduces new considerations from a surgical standpoint, including long-term valve durability, coronary access after implantation, and the management of younger patients who may require multiple valve interventions over their lifetime [8,9].

Mitral stenosis (MS), although less prevalent in Western countries than AS, remains clinically relevant, particularly in elderly patients with extensive mitral annular calcification (MAC) or in populations where rheumatic heart disease persists. The coexistence of AS and MS presents unique diagnostic and therapeutic challenges. Hemodynamic interactions between the two stenotic lesions may lead to underestimation of disease severity, altered symptom presentation, and increased procedural risk [3]. Patients with combined AS–MS often represent advanced valvular degeneration and frequently exhibit atrial fibrillation (AF), pulmonary hypertension, right ventricular dysfunction, and extensive annular calcification, all of which increase operative complexity and perioperative risk [3,11,12]. Despite major advances in both surgical and transcatheter valve therapies, patients with concomitant AS and MS remain underrepresented in randomized trials. As a result, contemporary guideline recommendations for this population are largely based on expert consensus and observational data [2,3,12]. Double-valve surgery remains the definitive treatment for patients with acceptable operative risk, but it is associated with higher morbidity and mortality compared with isolated valve procedures, reflecting the cumulative operative burden and advanced disease substrate typical of this population [11,12]. Conversely, transcatheter and hybrid strategies have emerged as potential alternatives for high-risk patients, although supporting evidence is limited and long-term outcomes remain uncertain [12,13,14].

Against this backdrop, a comprehensive and clinically focused synthesis of current evidence is needed. The present narrative review aims to summarize contemporary treatment strategies for isolated AS and concomitant AS–MS. Emphasis is placed on guideline-directed management, operative decision-making, emerging transcatheter and hybrid approaches, the central role of the multidisciplinary Heart Team in optimizing patient outcomes, and the need for updated, specific guidelines.

## 2. Methodology

The format of a narrative review is the most suitable because of the heterogeneous nature of the current literature on this topic. The narrative review is the best tool to provide a complete synopsis and describe the current status. A systematic literature search was conducted through the PubMed database to identify relevant papers published between 1 January 2015 and 31 December 2025. We selected the 2015–2025 interval to include all recent and impactful articles. We decided to use the PubMed database as it is the most widely accessible and comprehensive source for biomedical literature, ensuring transparency and reproducibility of our approach. The following advanced search query were used: (“Aortic Valve Stenosis”[Majr]) AND (“Aortic Valve Replacement”[MeSH] OR “Transcatheter Aortic Valve Replacement”[MeSH]) AND guideline[Publication Type]; (“Aortic Valve Stenosis”[MeSH]) AND (“Mitral Valve Stenosis”[MeSH]) AND (surgical[Title/Abstract] OR percutaneous[Title/Abstract] OR transcatheter[Title/Abstract]).

Inclusion criteria comprised review articles, descriptive cohort studies, and case reports relevant to the topics identified through the previously listed queries. Exclusion criteria were duplicates, non-English articles, and studies clearly outside the scope of this review. We initially found 102 articles in total. As a first step, we read the titles of each study and selected the ones that referred to our topics. As a second step, we read abstracts to select the potentially interesting papers. The latter were carefully and thoroughly read (as a third step) to select the most appropriate articles for inclusion. A stepwise screening process was applied, whereby studies were progressively selected according to the thematic relevance of each individual article to the review question. The articles’ references were reviewed to be included accordingly. To avoid bias, the search and selection process was carried out by two authors independently (K.M., S.P.); the process was thoroughly supervised by a third author (A.R.). Any disagreements between the two independent reviewers during study selection and quality assessment were resolved through discussion. When consensus could not be reached, the senior supervisor (A.R.) was consulted to make the final decision. A reflexive approach was adopted by the authors to ensure transparency and awareness of potential interpretive bias. The literature search and selection were performed between January and February 2026. At the end of our search and selection process, we decided to include 38 studies in our narrative review.

## 3. Pathophysiology and Hemodynamics

### 3.1. Pathophysiology of Isolated Aortic Stenosis

AS is a progressive valvular disorder characterized by LVOTO due to structural and functional deterioration of the aortic valve. In contemporary Western populations, the dominant etiology is degenerative calcific disease, which primarily affects elderly patients and shares pathophysiological mechanisms with atherosclerosis [4].

Progressive leaflet thickening, fibrosis, and calcification lead to reduced systolic excursion and a gradual decline in effective aortic valve area. As valvular obstruction worsens, the left ventricle is subjected to chronic pressure overload [4]. In patients with severe AS, secondary tricuspid regurgitation (TR) may represent an advanced stage with myocardial fibrosis and stiffness, affecting mortality. It should be noted that from a diagnostic perspective, thermodilution-derived cardiac output can be erroneously low, which ultimately can result in an underestimation of aortic valve area and overestimation of AS severity [15].

Delayed referral for intervention may result in irreversible myocardial damage, such that symptomatic and functional improvement remains limited despite technically successful valve replacement [4,8]. Hemodynamically, severe AS is classically defined by an aortic valve area ≤ 1.0 cm^2^, a mean transvalvular gradient ≥ 40 mmHg, or a peak aortic jet velocity ≥ 4.0 m/s [1,2,3]. However, discordant grading patterns are increasingly recognized, particularly in elderly patients with reduced stroke volume. Low-flow, low-gradient AS with preserved or reduced left ventricular ejection fraction represents a diagnostically challenging subgroup that requires careful multimodality assessment and clinical correlation [4].

### 3.2. Pathophysiology of Mitral Stenosis

MS is characterized by obstruction to left ventricular inflow, resulting in elevated left atrial pressure, pulmonary venous congestion, and progressive pulmonary hypertension. While rheumatic heart disease remains the predominant cause globally, degenerative MS related to extensive MAC has become increasingly prevalent in elderly patients in high-income regions [16]. Degenerative MS differs fundamentally from rheumatic disease: calcification extends from the annulus into the leaflets and subvalvular apparatus, leading to restricted leaflet motion without commissural fusion [17].

Chronic elevation of left atrial pressure promotes atrial dilation, AF, and thromboembolic risk. Progressive pulmonary hypertension increases right ventricular afterload, ultimately leading to right ventricular dysfunction and right-sided heart failure. These pathophysiological changes significantly influence surgical risk and long-term prognosis [18].

### 3.3. Concomitant Aortic and Mitral Stenosis

The coexistence of AS and MS introduces complex and often counterintuitive hemodynamic interactions that complicate both diagnosis and management. MS limits left ventricular preload, resulting in reduced stroke volume and lower transaortic gradients. This phenomenon may lead to underestimation of AS severity when relying solely on Doppler-derived gradients [3,11,19].

Conversely, severe AS may mask the clinical impact of MS by limiting forward flow. Following relief of aortic outflow obstruction—either surgically or via transcatheter intervention—increased stroke volume may unmask or exacerbate trans-mitral gradients, revealing clinically significant MS that was previously underestimated. This interaction explains why some patients experience persistent or worsening symptoms after isolated treatment of AS [2,14]. Combined AS and MS exert a compounded effect on biventricular remodeling and promote progressive pulmonary hypertension. Advanced pulmonary hypertension and ventricular failure significantly increase perioperative mortality and complicate postoperative management [3].

### 3.4. Implications for Surgical and Transcatheter Decision-Making

Understanding the pathophysiological interplay between AS and MS is fundamental to selecting the optimal treatment strategy (Table 1). In isolated AS, relief of LVOTO typically results in rapid hemodynamic improvement and symptomatic relief. In contrast, patients with combined disease may require simultaneous or staged correction of both lesions to achieve meaningful clinical benefit. Comprehensive anatomical and physiological assessment—including echocardiography, computed tomography (CT), and, when necessary, invasive hemodynamics—is essential to guide operative planning [1,2,3].

## 4. Diagnostic Assessment

### 4.1. Transthoracic Echocardiography

Transthoracic echocardiography (TTE) remains the first-line imaging modality for the evaluation of both AS and MS and is recommended as the initial diagnostic test in all patients with suspected valvular heart disease [1,2]. TTE allows comprehensive assessment of valve morphology, transvalvular gradients, valve areas, ventricular size and function, and pulmonary pressures, forming the cornerstone of initial diagnostic and therapeutic decision-making.

In isolated AS, standard echocardiographic parameters include peak aortic jet velocity, mean transvalvular gradient, and calculated aortic valve area using the continuity equation. These parameters constitute the basis of guideline-defined severity classification and inform surgical timing and procedural selection. Assessment of left ventricular wall thickness, diastolic function, and systolic performance provides critical adjunctive information relevant to operative risk stratification and myocardial reserve. In MS, echocardiographic evaluation focuses on mitral valve area (derived by planimetry or pressure half-time), mean transmitral gradient, left atrial size, pulmonary artery systolic pressure, and the presence of associated mitral regurgitation. In degenerative MS, extensive annular and leaflet calcification frequently limits the accuracy of planimetric and pressure half-time measurements, necessitating careful integration of multiple echocardiographic and hemodynamic parameters to avoid under- or overestimation of disease severity [1,2,3,13,20].

In patients with combined AS and MS, TTE interpretation is particularly challenging. Reduced left ventricular preload due to MS results in lower stroke volume and underestimation of AS severity. Conversely, severe AS may limit forward flow across the mitral valve, masking the functional severity of MS [3]. These hemodynamic interactions increase the prevalence of discordant findings, such as low-gradient AS. In such cases, reliance on a single echocardiographic parameter is insufficient, and comprehensive evaluation incorporating valve morphology, flow indices, ventricular function, and clinical presentation is mandatory [3,11]. From a surgical standpoint, misclassification of valvular severity may result in inappropriate isolated valve intervention. Persistent symptoms following isolated aortic valve replacement in patients with unrecognized clinically significant MS underscore the consequences of incomplete preoperative assessment and highlight the need for meticulous multimodality evaluation [2,11,20].

Stress echocardiography may be useful in selected patients with discordant AS severity or equivocal symptoms. In low-flow, low-gradient AS, dobutamine stress echocardiography allows differentiation between true severe AS and pseudo-severe disease by assessing contractile reserve and evaluating changes in aortic valve area and transvalvular gradients under conditions of increased flow [3].

### 4.2. Transesophageal Echocardiography

Transesophageal echocardiography (TEE) plays a pivotal role in complex or combined valvular disease. TEE provides superior spatial resolution for assessment of mitral valve anatomy, annular calcification, subvalvular involvement, and commissural morphology, all of which are critical for determining the feasibility of valve repair, replacement, or percutaneous intervention. In patients with combined AS–MS, TEE is particularly valuable when transthoracic echocardiography findings are inconclusive or discordant. Three-dimensional TEE enables accurate mitral valve planimetry and detailed characterization of calcific burden and leaflet mobility, factors that directly influence surgical strategy, operative risk, and technical complexity. From an intraoperative perspective, TEE is indispensable for confirming preoperative findings, guiding surgical repair or replacement, and assessing immediate procedural results. Intraoperative detection of residual transvalvular gradients, paravalvular leak, or ventricular dysfunction permits prompt correction and is associated with improved procedural safety and early surgical outcomes [2,3,11].

### 4.3. Computed Tomography

Multidetector CT scan has become an integral component of contemporary valvular assessment. In AS, CT enables accurate quantification of aortic valve calcification, which is particularly valuable in patients with low-gradient disease or equivocal echocardiographic findings. CT-derived aortic valve calcium scores provide flow-independent confirmation of severe AS and support clinical decision-making when Doppler-derived parameters are discordant.

CT imaging is also essential for procedural planning in both surgical and transcatheter interventions. Detailed assessment of annular dimensions, leaflet and annular calcification, coronary ostial height, and aortic root anatomy informs prosthesis selection and reduces the risk of procedural complications, including annular rupture, coronary obstruction, and paravalvular leak [3,11,21]. In patients with concomitant AS–MS, CT allows precise MAC evaluation, which represents a key determinant of surgical complexity and feasibility of transcatheter mitral interventions [11]. The extent, distribution, and depth of calcification directly influence operative strategy, including the need for annular decalcification, patch reconstruction, or consideration of hybrid or transcatheter approaches.

### 4.4. Invasive Hemodynamic Assessment

Cardiac catheterization remains an important adjunctive diagnostic tool when non-invasive imaging is inconclusive or yields conflicting results. Direct measurement of transvalvular gradients, intracardiac pressures, and cardiac output can help clarify lesion severity in complex cases, particularly when combined stenotic lesions significantly alter flow dynamics [1,2].

Invasive assessment may be especially useful in patients with suspected low-flow states, severe pulmonary hypertension, or advanced ventricular dysfunction, where echocardiographic parameters may underestimate true disease severity. Simultaneous left ventricular and aortic pressure measurements can improve accuracy in grading AS, while pulmonary artery and left atrial pressure assessment provides valuable insight into the hemodynamic consequences of MS. However, catheter-derived gradients remain flow-dependent and must be interpreted with caution in the presence of concomitant MS. Reduced left ventricular preload due to MS may result in deceptively low aortic gradients, potentially leading to underestimation of AS severity if invasive data are interpreted in isolation [1,2,3,22,23].

## 5. Surgical Treatment

### 5.1. Surgical Aortic Valve Replacement in Isolated Aortic Stenosis

SAVR remains the reference standard for patients with symptomatic severe AS who are at low or intermediate operative risk (Table 2). Both American and European guidelines assign SAVR a Class I recommendation in younger patients, those with low surgical risk scores, and individuals requiring concomitant cardiac surgery [1,2]. Large contemporary surgical series and registry analyses confirm excellent early and long-term outcomes following SAVR, with sustained symptomatic relief and favorable survival compared with age-matched populations [1,2,5]. SAVR provides immediate and complete relief of LVOTO. Direct visualization allows meticulous excision of heavily calcified leaflets and controlled annular decalcification, minimizing paravalvular leak and optimizing prosthesis seating. These advantages are particularly relevant in patients with extensive annular calcification or small annular dimensions, where transcatheter approaches may be associated with higher residual gradients or prosthesis–patient mismatch [5].

When performing left heart valve surgery, current guidelines recommend additional tricuspid valve surgery for concomitant TR because it has been shown to prevent worsening of TR and facilitate reverse remodeling of the RV and improvement of functional status without increasing operative risk. Concomitant tricuspid valve surgery is indicated even for mild TR in the presence of tricuspid annular dilation or signs of right heart failure, as it may worsen later after left heart valve surgery and possibly cause poor clinical outcomes [15].

Full median sternotomy remains the most versatile approach and is preferred in patients requiring complex anatomy correction or multiple concomitant procedures. Minimally invasive SAVR approaches may be considered in selected patients at experienced centers; however, patient selection and institutional expertise remain critical determinants of safety and outcomes, as emphasized by contemporary guideline documents [1,2]. Ministernotomy plays an important role in contemporary SAVR and is expected to become even more relevant in the future [24].

### 5.2. Double-Valve Surgery for Concomitant Aortic and Mitral Stenosis

In patients with concomitant AS and MS, combined surgical replacement provides definitive treatment of both obstructive lesions and remains the reference standard when operative risk is acceptable, as supported by contemporary European guidelines and surgical outcome studies [12]. Isolated correction of one valve may unmask or exacerbate the severity of the remaining lesion [3]. Double-valve surgery is associated with increased operative complexity, longer cardiopulmonary bypass times, and higher perioperative risk compared with isolated valve procedures. Contemporary observational studies and surgical registries consistently report higher rates of early morbidity and mortality, particularly among elderly patients and those with advanced comorbidities [12]. Nevertheless, long-term symptomatic improvement and acceptable survival can be achieved in carefully selected patients, underscoring the importance of individualized risk assessment and multidisciplinary Heart Team evaluation [1,2].

Extensive MAC represents a major technical challenge in this setting. Surgical strategies may include partial annular decalcification with patch reconstruction, supra-annular valve implantation, or, in selected high-risk patients, hybrid approaches incorporating transcatheter technologies. Each strategy requires individualized risk–benefit assessment, meticulous preoperative imaging, and advanced surgical expertise [12].

### 5.3. Outcomes of Surgical Management

Contemporary surgical series demonstrate excellent long-term outcomes following isolated SAVR, with low rates of structural valve deterioration and durable improvements in functional status and survival [4,5,6]. Advances in myocardial protection, perioperative management, and patient selection have contributed to consistently decreased operative mortality in low- and intermediate-risk patients. Outcomes following double-valve surgery are less favorable but remain acceptable in carefully selected patients (Table 3). Advanced age, impaired ventricular function, pulmonary hypertension, frailty, and extensive annular calcification are consistently associated with increased perioperative risk and reduced long-term survival [12,13].

A retrospective study by Husso et al. showed a higher 30-day mortality in patients who underwent double-valve surgery for concomitant AS–MS compared with patients who underwent single-valve surgery or those who underwent double valve surgery for AS-MR. This reflected both the extreme rarity of the cases and the advanced heart disease. Preoperative increased PAP and decreased GFR were predictive for lower survival, as well as lengthy operations and postoperative low-output syndrome [25].

While the type of bioprosthesis has shown minimal differences in durability, the bioprosthesis degeneration incidence has been more significant in the mitral position rather than the aortic position [26].

**Figure 1:**
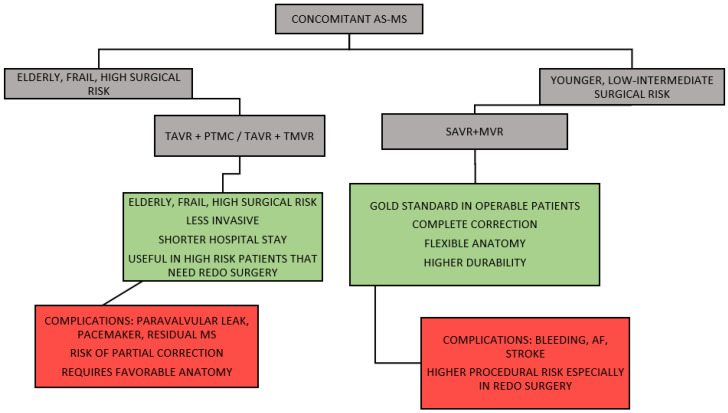
Concomitant AS–MS Indications. AS, aortic stenosis; AF, atrial fibrillation; MS, mitral stenosis; MVR, Mitral valve replacement; SAVR, surgical aortic valve replacement; TAVR, transcatheter aortic valve replacement; TMVR, transcatheter mitral valve replacement; PTMC, percutaneous transvenous mitral commissurotomy.

These findings reinforce the importance of early referral, comprehensive preoperative assessment, and individualized operative planning within a multidisciplinary Heart Team (Figure 1) [1,2].

## 6. Transcatheter and Hybrid Procedures

### 6.1. Transcatheter Aortic Valve Replacement in Isolated Aortic Stenosis

TAVR has become an established alternative to SAVR in patients with symptomatic severe AS across a broad spectrum of surgical risk (Table 4). Contemporary randomized controlled trials and large registry analyses have demonstrated non-inferior—and in carefully selected populations, superior—early outcomes compared with surgery, particularly with respect to early mortality, stroke, and recovery time (Table 5) [5,7,8,27]. Current international guidelines endorse TAVR as a Class I recommendation in patients aged ≥70 years or in those with increased surgical risk, while emphasizing the importance of anatomical suitability, procedural feasibility, and life expectancy when selecting candidates [1,2]. Meticulous preprocedural imaging is essential to assess annular dimensions, coronary ostial height, leaflet and annular calcification distribution, and vascular access, as these factors directly influence procedural safety, complication risk, and long-term outcomes. Despite excellent short- and mid-term clinical results, concerns remain regarding the long-term durability of transcatheter valves, particularly in younger patients with longer anticipated survival. Available data suggest acceptable structural valve performance of contemporary transcatheter prostheses up to approximately 8–10 years; however, durability beyond this time horizon remains incompletely defined [5,7,8,27,28,29]. These considerations reinforce the continued role of SAVR in younger, low-risk patients and underscore the importance of lifetime valve management planning within a multidisciplinary Heart Team framework [1,2,30].

It has been suggested that TR improves in 15% to 60% of cases and normalization of RV function was observed in more than one-half of the patients after TAVR, which seems to be better than in patients undergoing SAVR [15].

### 6.2. Transcatheter Mitral Valve Interventions in the Setting of Mitral Stenosis

In contrast to AS, transcatheter treatment options for MS remain limited. Percutaneous mitral balloon valvotomy (PMBV) continues to represent the preferred intervention for selected patients with rheumatic MS and favorable valve morphology, as endorsed by contemporary international guidelines [1,2]. However, PMBV is unsuitable for patients with significant leaflet or annular calcification, advanced subvalvular involvement, or concomitant severe AS—conditions that are frequently encountered in elderly patients. Transcatheter mitral valve replacement (TMVR) in native MS remains investigational and is largely restricted to highly selected patients. Extensive MAC, frequently present in elderly patients with concomitant AS, poses major technical challenges and is associated with a high risk of LVOTO, device embolization, and early mortality. These limitations underscore the continued importance of operative MVR in patients with significant MS who are suitable surgical candidates, particularly when combined correction of AS is required [13]. TMVR has emerged as an alternative to redo surgery, and preliminary data have shown no significant difference in postoperative mean mitral gradient, 30-day mortality, and 1-year mortality when compared with redo surgery [32].

### 6.3. Transcatheter and Hybrid Approaches in Combined Aortic and Mitral Stenosis

Hybrid procedures (combined surgical and transcatheter interventions) have emerged as potential alternatives for carefully selected high-risk patients with concomitant AS and MS who are not suitable candidates for conventional double-valve surgery (Table 6). These approaches may include TAVR combined with percutaneous mitral balloon valvotomy in rheumatic disease, or staged TAVR followed by transcatheter mitral interventions in selected cases with prohibitive surgical risk [1,2]. Hybrid procedures aim to reduce operative trauma, cardiopulmonary bypass exposure, and perioperative risk. However, the supporting evidence is limited to small observational studies and case series, characterized by heterogeneous patient selection, variable procedural sequencing, and short follow-up durations. Reported clinical outcomes remain inconsistent, and robust long-term durability data are lacking [13,31].

A systematic review from Ando et al. showed that the main complications associated with both TAVR and TMVR were associated with several perioperative complications, including ostial coronary occlusion, higher mean gradient post-TAVR, significant paravalvular leak, valve malposition, and LV obstruction. The most common strategy for combined valve replacement was to treat the aortic valve first, followed by the mitral valve. Aortic and mitral annuli are anatomically contiguous, bridged by the aorto-mitral fibrous curtain, and function interdependently. When the mitral valve is treated first, there is a concern for obstructing the path to deploying a new aortic valve via the transapical approach. Another concern is that the struts of the biological surgical mitral valve may cause the transcatheter-deployed valve in the aortic position to be displaced toward the aorta, resulting in embolization or significant paravalvular regurgitation. For double transcatheter valve replacement, most of the cases were treated in a simultaneous session as opposed to TAVR/TAViV (transcatheter aortic valve in valve) + TMVR cases since TAVR will unlikely affect the degree of MS. Rather, even worsening cases of MS have been reported. In addition, because many of the cases were performed through transapical access, performing in a separate session would merely increase the risk of further cardiac damage, bleeding, and vascular risk through sheath cannulation to the left ventricular apex [33].

### 6.4. Limitations of Transcatheter Therapies

While transcatheter therapies offer less invasive treatment options, they are associated with specific limitations and complications that must be carefully weighed against surgical alternatives. These include paravalvular regurgitation, an increased need for permanent pacemaker implantation, vascular access complications, and challenges related to coronary access following TAVR [5,7]. In patients with combined valvular disease, incomplete correction of concomitant mitral pathology following isolated TAVR may result in persistent symptoms, ongoing adverse cardiac remodeling, and the need for subsequent interventions [3,30]. Furthermore, prior TAVR may complicate future surgical procedures due to altered aortic root anatomy, prosthesis-related constraints, and increased technical complexity during a subsequent surgery [15,27]. These considerations reinforce the importance of long-term treatment planning and underscore the central role of the cardiac surgeon in guiding therapeutic strategy, even when transcatheter approaches are selected as part of the initial management plan [1,2].

## 7. Discussion

The management of isolated AS and concomitant AS–MS has undergone substantial evolution over the past decade, driven by advances in surgical techniques, transcatheter technologies, and an increasing emphasis on individualized, guideline-directed care [1,2]. These developments have expanded the therapeutic armamentarium while simultaneously increasing the complexity of clinical decision-making, particularly in patients with multivalvular disease, where anatomical heterogeneity, comorbidity burden, and long-term durability considerations must be carefully balanced [1,2].

### 7.1. Isolated Aortic Stenosis: Surgery Versus Transcatheter Therapy

In isolated AS, both SAVR and TAVR are now firmly established treatment options. Contemporary American and European guidelines emphasize patient age, surgical risk, anatomical suitability, and life expectancy as the primary determinants of treatment selection [1,2]. While randomized trials and large registry data demonstrate excellent short-term outcomes with TAVR across multiple surgical risk strata, surgery continues to offer distinct advantages in younger patients, those with longer anticipated survival, and individuals requiring concomitant cardiac procedures [4,5]. SAVR provides predictable and complete relief of LVOTO, durable valve performance, and the ability to directly address annular pathology and associated cardiac disease. These attributes remain particularly relevant in patients with small annular dimensions, extensive valvular or annular calcification, or complex coronary anatomy, where transcatheter approaches may be limited or associated with increased procedural risk [7,10]. Consequently, despite the expansion of TAVR indications, surgery retains a major role in contemporary AS management.

In patients with high surgical risk, TAVR is more cost-effective than SAVR, and TAVR + percutaneous coronary intervention (PCI) is more cost-effective than SAVR + coronary artery bypass grafting (CABG). This benefit is linked to lower procedural and in-hospital death with interventional treatment (TAVR or TAVR + PCI) in high-risk patients. However, in patients without high surgical risk, SAVR is more cost-effective than TAVR, and SAVR + CABG is more cost-effective than TAVR + PCI. While other studies showed similar hospitalization costs between TAVR and SAVR, in our study, TAVR hospitalization costs were over EUR 20,000 higher than those for SAVR, since the difference in procedural costs was also maintained in the total hospitalization cost [34].

### 7.2. Concomitant Aortic and Mitral Stenosis: Persisting Surgical Dominance

The management of concomitant AS–MS remains significantly more challenging and less standardized than that of isolated AS. Hemodynamic interactions between the two valves complicate accurate severity assessment, procedural timing, and therapeutic sequencing, while the evidence base guiding management is substantially more limited than for single-valve disease [3]. Combined surgical correction remains the most definitive and comprehensive treatment strategy for patients with acceptable operative risk. Double-valve surgery allows simultaneous relief of both obstructive lesions, avoids residual valvular obstruction, and reduces the likelihood of persistent or recurrent symptoms following isolated intervention [14]. Although operative risk is higher than for isolated valve replacement, contemporary surgical series demonstrate acceptable early and mid-term outcomes in carefully selected patients, particularly when intervention is undertaken before the development of advanced ventricular dysfunction or severe pulmonary hypertension [1,2].

### 7.3. Role and Limitations of Transcatheter and Hybrid Strategies

Transcatheter and hybrid approaches have emerged as potential alternatives for selected high-risk patients with combined valvular disease who are deemed unsuitable for conventional surgery. TMVR in native MS remains investigational and is associated with substantial procedural risk, especially in the presence of severe MAC [19]. Strategies such as TAVR combined with percutaneous mitral balloon valvotomy, or staged transcatheter interventions targeting each valve sequentially, may offer symptomatic improvement with reduced procedural invasiveness in carefully selected cases [31].

Despite significant progress in transcatheter valve therapies, such as TAVR, trans-catheter edge-to-edge repair, and TMVR, there is limited data on the outcomes of combined multi-valvular transcatheter interventions involving trans-catheter edge-to-edge repair or TMVR during the same hospitalization as TAVR. In patients undergoing transcatheter treatment for both aortic and mitral disease, the optimal approach, staged or concomitant, remains unclear. Concomitant transcatheter implantation of TAVR for AS followed by a TMVR in MS is a feasible option for patients at high surgical risk. Few cases of concomitant transcatheter interventions have been documented and have been reserved for high-risk patients deemed inoperable [35,36,37].

Transcatheter and hybrid strategies have expanded therapeutic options for elderly and high-risk patients and patients who cannot undergo redo surgery. However, this remains specific and limited cases to be discussed in a multi-disciplinary setting and are not yet well indicated by American and European guidelines.

These limitations underscore that percutaneous and hybrid approaches should be reserved for exceptional circumstances and undertaken only in experienced centers, within a multidisciplinary Heart Team framework, with the requisite expertise to manage complex perioperative and transcatheter-related complications.

### 7.4. Heart Team Approach and Lifetime Valve Management

A recurring theme across contemporary guideline documents is the central role of the multidisciplinary Heart Team in guiding management decisions [1,2]. This collaborative approach is particularly critical in patients with combined AS and MS, in whom anatomical complexity, comorbidity burden, procedural risk, and long-term therapeutic strategy must be carefully integrated into individualized treatment decisions. Accurate diagnosis and severity assessment are central both for correct indication and optimal management. Transcatheter and hybrid strategies should be viewed not as competitors to surgery but as complementary tools. The concept of lifetime valve management has gained increasing prominence, particularly as transcatheter therapies are extended to younger and lower-risk patient populations [3,6,8]. Studies show structural valve dysfunction is significantly greater for SAVR compared with TAVR, mainly due to higher mean valve gradients present shortly after the procedure [8]. However, treatment decisions should not be driven solely by short-term procedural outcomes, but must also account for valve durability, the feasibility and complexity of future interventions, and the cumulative risk associated with repeated valve procedures over the patient’s lifetime. Within this framework, surgery continues to play a foundational role—not only as a definitive primary treatment strategy, but also as an essential component in the management of failed or degenerated transcatheter valve therapies.

### 7.5. Knowledge Gaps and Future Directions

Despite substantial progress in the treatment of AS, important gaps remain in the management of both isolated AS and concomitant AS–MS. Long-term durability data for transcatheter aortic valve prostheses beyond the first decade remain limited, particularly in younger patients with extended life expectancy [3,6,8]. Future research should prioritize long-term durability for the transcatheter aortic valve, considering its current use in younger patients. In addition, robust comparative data evaluating outcomes of surgical versus hybrid or staged transcatheter strategies in patients with combined valvular disease are lacking, as current evidence is largely derived from small observational studies and registry analyses [13,14]. Considering the heterogeneity of the multivalvular disease, future research should prioritize prospective registries and collaborative multi-center studies in order to further refine patient selection, improve procedural safety, and expand therapeutic options, standardizing them in a wider global context. Furthermore, the treatment of high-risk patients affected by infective endocarditis has grown interest in exploring alternative approaches such as sutureless valves and TAVR, even though few reports and case series have been reported so far. Future larger studies with a long-term follow-up are warranted to evaluate the safety and durability of these approaches in such particular cases [38].

Hybrid and percutaneous approaches for concomitant AS–MS treatment remain limited to high-risk patients for surgical repair, mainly patients with previous cardiac surgery in need of redo surgery. The data on post-procedural outcome remains limited, considering the importance of the percutaneous approach as a therapeutic option, due to only a few cases being described. The need to create multi-center studies and registries would create a significant role in choosing the best therapeutic option in consideration of patient characteristics.

Guidelines must become more rigorous and explicitly detailed, considering the anticipated increase in these cases and procedures. Current recommendations often lack sufficient granularity across all treatment modalities, especially regarding percutaneous approaches. Updated guidelines should clearly define patient selection criteria, timing of intervention, and procedural strategies to ensure optimal, evidence-based, and standardized care for all patients.

### 7.6. Limitations

This narrative review has several inherent limitations. We preferred to use a single database (PubMed); despite its several advantages, a single database may limit our results. As a non-systematic synthesis, it is subject to selection and interpretive bias despite the use of a structured literature search. The evidence base for concomitant AS and MS is limited and heterogeneous, as patients with multivalvular disease are underrepresented in randomized trials and most available data derive from observational studies and registries. In addition, many included studies reflect earlier generations of transcatheter devices, and long-term durability data—particularly beyond 8–10 years—remain limited, especially for younger patients. Finally, evolving technologies, center-specific expertise, and patient-level factors such as frailty and comorbidity may influence outcomes and limit the generalizability of available evidence.

## 8. Conclusions

The contemporary management of valvular heart disease has evolved into an increasingly nuanced and multidisciplinary field, particularly in the setting of isolated AS and concomitant AS and MS. SAVR remains the cornerstone of durable treatment for isolated AS. In patients with concomitant AS and MS, management is more complex and remains insufficiently addressed by randomized clinical trials. Combined surgical valve replacement provides definitive correction of both lesions and remains the reference standard. However, the increased morbidity associated with double-valve surgery necessitates careful patient selection, optimal timing of intervention, and individualized operative planning. Transcatheter and hybrid strategies have expanded therapeutic options for elderly and high-risk patients, although this remains limited to specific cases to be discussed in a multi-disciplinary setting and is not yet well indicated by American and European guidelines.

Although there is not yet a well-standardized approach for treating concomitant AS–MS, the expansion of therapeutic strategies, including percutaneous and hybrid, has expanded the treatment possibilities in high-risk patients. The multidisciplinary Heart Team is crucial for patients’ selection and indication; the concept of lifetime valve management is also paramount. Future high-quality data should lead to more specific guidelines to target a patient-customized approach.

Due to the limited cases, we suggest that creating multi-center registries would be crucial for reaching a global consensus on the best way to treat the concomitant AS–MS.

## Figures and Tables

**Table 1:** Contemporary Guidelines for Isolated Aortic Stenosis and Concomitant with Mitral Stenosis.

Clinical Scenario	ACC/AHA Guidelines	ESC/EACTS Guidelines	Surgical Perspective
Symptomatic severe isolated AS	SAVR or TAVR recommended depending on age, risk, anatomy	SAVR favored in younger, low-risk patients; TAVR in older/high-risk	SAVR preferred in patients with long life expectancy or need for concomitant procedures
Asymptomatic severe AS	Intervention if LV dysfunction, very severe AS, or rapid progression	Similar criteria with emphasis on LV function and exercise testing	Early surgery may prevent irreversible myocardial remodeling
Low surgical risk AS	TAVR acceptable in selected patients	SAVR preferred in younger patients	Durability and lifetime management favor surgery
AS with concomitant MS	Individualized Heart Team decision	Individualized; limited specific guidance	Double-valve surgery remains reference standard when feasible
Multivalvular disease	Heart Team evaluation recommended	Heart Team evaluation mandatory	Surgical correction allows definitive multivalvular treatment
Frailty/high operative risk	TAVR favored	TAVR favored	Hybrid strategies considered in select cases

AS, aortic stenosis; LV, left ventricle; MS, mitral stenosis; SAVR, surgical aortic valve replacement; TAVR, transcatheter aortic valve replacement.

**Table 2:** Surgical Aortic Valve Replacement: Key Operative Considerations in Isolated Aortic Stenosis.

Domain	Surgical Considerations	Clinical Implications
Annular calcification	Requires controlled and selective decalcification to prevent annular rupture or conduction injury	Influences prosthesis choice and operative risk
Myocardial protection	Hypertrophied ventricles require optimized cardioplegia delivery and myocardial protection strategies	Reduces risk of perioperative low cardiac output syndrome
Prosthesis selection	Mechanical vs. bioprosthetic valve choice based on age, life expectancy, anticoagulation tolerance	Impacts durability, reintervention strategy, and lifetime management
Annular size	Small annulus may require enlargement techniques	Prevents patient–prosthesis mismatch
Concomitant pathology	Coronary artery disease, ascending aorta disease, or additional valve lesions can be addressed simultaneously	Major advantage over isolated transcatheter approaches
Intraoperative imaging	Mandatory trans-esophageal echocardiography	Confirms valve seating, gradients, and absence of paravalvular leak
Long-term outcomes	Excellent durability and sustained hemodynamic performance	Remains benchmark therapy in low–intermediate risk patients

**Table 3:** Comparison of Isolated SAVR and Double-valve Surgery for AS and MS.

Parameter	Isolated SAVR	Double-Valve Surgery (AS–MS)
Procedural complexity	Moderate	High
Cardiopulmonary bypass time	Short to moderate	Prolonged
Cross-clamp duration	Short	Long
Technical challenges	Annular calcification	Severe MAC, sub valvular disease
Perioperative morbidity	Low	Moderate to high
Risk of conduction disturbances	Moderate	Increased
Definitiveness of treatment	Single lesion corrected	Complete multivalvular correction
Long-term durability	Excellent	Excellent in selected patients
Ideal candidates	Low–intermediate risk, isolated AS	Acceptable-risk patients with severe AS–MS

AS, aortic stenosis; MAC, mitral annular calcification; MS, mitral stenosis; SAVR, surgical aortic valve replacement.

**Table 4:** Key Decision Patterns for TAVR vs. SAVR.

Patient Characteristic	TAVR Preferred	SAVR Preferred
Age	>70 years	<70 years
Life expectancy	<10 years	>10 years
Surgical risk	High/prohibitive risk	Low risk
Frailty	Significant	Fit
Comorbidities (chronic kidney disease, chronic obstructive pulmonary disease, severe vasculopathy, diabetes)	Severe comorbidities	Minimal comorbidity
Aortic valve	If favorable anatomy	Bicuspid valve in younger patients
Annulus size/access	Suitable	Unfavorable
Coronary artery disease	PCI feasible	CABG needed
MS, MR, TR	Mild disease	Severe mitral/tricuspid disease
Aortic root/ascending aorta	No major aortopathy	Aneurysm, root dilatation
Need for anticoagulation	Avoid anticoagulation	Already requires anticoagulation
Previous cardiac surgery	Favor TAVR	
Conduction system risk	Higher pacemaker risk	Lower pacemaker risk
Bleeding risk	Lower	Higher

MR, mitral regurgitation; MS, mitral stenosis; SAVR, surgical aortic valve replacement; TAVR, transcatheter aortic valve replacement; TR, tricuspid regurgitation.

**Table 5:** Key Contemporary Trials and Registries Informing Surgical and Transcatheter Treatment of Aortic Stenosis.

Study/Registry	Population	Comparison	Key Findings	Surgical Relevance
PARTNER 2 & 3 [7,10,28,31]	Intermediate and low-risk AS	TAVR vs. SAVR	Noninferior early outcomes	Durability and pacemaker risk remain concerns
SURTAVI [28]	Intermediate-risk AS	TAVR vs. SAVR	Similar mortality and stroke	Surgery superior for concomitant disease
NOTION [7,8,28]	Low-risk AS	TAVR vs. SAVR	Comparable mid-term outcomes	Structural valve durability under evaluation
STS/ACC TVT Registry [28]	Real-world TAVR	Observational	Expanding indications	Highlights complication management role of surgeons
Surgical registries [5,6,12,28]	Isolated and multivalvular surgery	Observational	Durable long-term outcomes	Reinforces SAVR and double-valve surgery as benchmarks

AS, aortic stenosis; SAVR, surgical aortic valve replacement; TAVR, transcatheter aortic valve replacement.

**Table 6:** Transcatheter and Hybrid Treatment Strategies in Isolated and Concomitant Valvular Stenosis.

Strategy	Primary Indications	Advantages	Key Limitations
Isolated TAVR	Severe AS, high or prohibitive surgical risk	Minimally invasive, rapid recovery	Pacemaker risk, durability uncertainty
TAVR with untreated MS	Severe AS with mild MS	Symptom improvement from AS relief	Persistent or worsened MS symptoms
Staged TAVR → MV intervention	Severe AS + moderate MS	Allows reassessment after AS correction	Multiple procedures, cumulative risk
Surgical MV + later TAVR	Severe MS with moderate AS	Definitive MV correction	Risk of interval AS progression
Hybrid surgical + transcatheter	High-risk multivalvular disease	Tailored approach	Requires advanced Heart Team coordination
TMVR (ViMAC)	Inoperable degenerative MS	Option for no-surgery candidates	High early mortality, LVOT obstruction

AS, aortic stenosis; LVOT, left ventricular outflow tract; MS, mitral stenosis; MV, mitral valve; TAVR, transcatheter aortic valve replacement, TMVR, transcatheter mitral valve replacement; ViMAC, valve-in-mitral annular calcification.

## Data Availability

No new data were created or analyzed in this study. Data sharing is not applicable.

## Abbreviations

AF	atrial fibrillation
AS	aortic stenosis
CABG	coronary artery bypass grafting
CT	computed tomography
LV	left ventricle
LVOT	left ventricular outflow tract
MAC	mitral annular calcification
MS	mitral stenosis
MR	mitral regurgitation
PCI	percutaneous coronary intervention
PMBV	percutaneous mitral balloon valvotomy
PVL	paravalvular leak
RV	right ventricle
SAVR	surgical aortic valve replacement
TAVR	transcatheter aortic valve replacement
TAViV	transcatheter aortic valve in valve
TEE	transesophageal echocardiography
TMVR	transcatheter mitral valve replacement
TR	tricuspid regurgitation
TTE	transthoracic echocardiography
ViMAC	valve-in-mitral annular calcification
